# From comorbidities of chronic obstructive pulmonary disease to identification of shared molecular mechanisms by data integration

**DOI:** 10.1186/s12859-016-1291-3

**Published:** 2016-11-22

**Authors:** David Gomez-Cabrero, Jörg Menche, Claudia Vargas, Isaac Cano, Dieter Maier, Albert-László Barabási, Jesper Tegnér, Josep Roca, on behalf of Synergy-COPD Consortia

**Affiliations:** 1 Department of Medicine, Karolinska Institutet, Unit of Computational Medicine, Stockholm, 171 77 Sweden; 2 Karolinska Institutet, Center for Molecular Medicine, Stockholm, 171 77 Sweden; 3 Department of Medicine, Unit of Clinical Epidemiology, Karolinska University Hospital, Solna, L8, 17176 Sweden; 4 Science for Life Laboratory, Solna, 17121 Sweden; 5 Center for Complex Networks Research and Department of Physics, Northeastern University, Boston, MA USA; 6 Institut d’Investigacions Biomèdiques August Pi i Sunyer (IDIBAPS), Hospital Clinic de Barcelona, Universitat de Barcelona, Barcelona, Spain; 7 Center for Biomedical Network Research in Respiratory Diseases (CIBERES), Madrid, Spain; 8 Biomax Informatics AG, Planegg, Germany; 9 Center for Cancer Systems Biology (CCSB) and Department of Cancer Biology, Dana-Farber Cancer Institute, Boston, MA USA; 10 Center for Network Science, Central European University, Budapest, Hungary; 11 Channing Division of Network Medicine, Department of Medicine, Brigham and Women’s Hospital, Harvard Medical School, Boston, MA USA; 12 Mucosal and Salivary Biology Division, King’s College London Dental Institute, London, UK

## Abstract

**Background:**

Deep mining of healthcare data has provided maps of comorbidity relationships between diseases. In parallel, integrative multi-omics investigations have generated high-resolution molecular maps of putative relevance for understanding disease initiation and progression. Yet, it is unclear how to advance an observation of comorbidity relations (one disease to others) to a molecular understanding of the driver processes and associated biomarkers.

**Results:**

Since Chronic Obstructive Pulmonary disease (COPD) has emerged as a central hub in temporal comorbidity networks, we developed a systematic integrative data-driven framework to identify shared disease-associated genes and pathways, as a proxy for the underlying generative mechanisms inducing comorbidity. We integrated records from approximately 13 M patients from the Medicare database with disease-gene maps that we derived from several resources including a semantic-derived knowledge-base. Using rank-based statistics we not only recovered known comorbidities but also discovered a novel association between COPD and digestive diseases. Furthermore, our analysis provides the first set of COPD co-morbidity candidate biomarkers, including IL15, TNF and JUP, and characterizes their association to aging and life-style conditions, such as smoking and physical activity.

**Conclusions:**

The developed framework provides novel insights in COPD and especially COPD co-morbidity associated mechanisms. The methodology could be used to discover and decipher the molecular underpinning of other comorbidity relationships and furthermore, allow the identification of candidate co-morbidity biomarkers.

**Electronic supplementary material:**

The online version of this article (doi:10.1186/s12859-016-1291-3) contains supplementary material, which is available to authorized users.

## Background

Chronic Obstructive Pulmonary Disease (COPD) is one of the five major chronic disorders in the WHO program for non-communicable diseases [1]. The disease is caused by inhalation of irritants (e.g. tobacco smoking or indoor pollution among others) in susceptible patients, and its prevalence is approximately nine percent of the adult population above 45 years of age. COPD is currently the fourth killer in Western countries and generates a major burden on healthcare systems worldwide [2].

Heterogeneity of both clinical manifestations [3] and disease progression [4] is a hallmark feature of COPD. Current clinical assessment of stable patients [5] relies on: (i) degree of lung function impairment (FEV_1_); (ii) symptoms score; (iii) risk for COPD exacerbations; iv) presence of co-morbidities; and, v) systemic effects of the disease [6–10]. While quantitative assessment of the first three criteria allows to allocate a given patient into one of the four disease severity stages (A to D) proposed by GOLD [5], a better understanding of co-morbid conditions is still needed for optimization of case management. There is also a need for a clear distinction between systemic effects of COPD (i.e. low-grade systemic inflammation and/or skeletal muscle dysfunction) [11–13] and some co-morbid conditions due to the descriptive nature of the reporting [14] with poor insight into underlying mechanisms of these phenomena [15]

Co-morbidities in COPD have well-known negative impact on patients’ prognosis and a close association with high use of healthcare resources [16, 17]. Consequently, the question is: Is COPD a risk factor for co-morbidities? The question has been has been recently answered negatively [18]. The authors suggest that risk factors, such as tobacco smoking and physical inactivity, explain the co-morbidity clustering seen in COPD patients, but COPD itself does not constitute a risk factor for co-morbidities. This finding, however, relies on research carried out in early COPD [19, 20].

The current study, in contrast, is based on a broader analysis and supports an alternative hypothesis indicating that patients with COPD may show higher risk for co-morbidities compared to non-COPD patients. We also hypothesized that abnormal regulation of key biological pathways in COPD patients, as well as shared underlying mechanisms, may explain certain clustering of co-morbid conditions often observed in the clinic. Moreover, the current hypothesis is consistent with the observation that disease co-occurrence also has a temporal component as shown in [21]. Therefore, uncovering the shared comorbidity-associated mechanisms should allow (*i*) case identification (e.g. to identify high risk patient with poor prognosis due to co-morbid conditions [22, 23]; (*ii*) define preventive strategies; and, (*iii*) explore novel therapeutic approaches [24]

In this study, we explore registries of approximately 13 M patients from the Medicare database [25], driven by two objectives. First, we analyzed if COPD individuals were at higher risk of being diagnosed with other diseases and if the association was modulated by age or gender.

Second, after identifying COPD co-morbidities, we performed a data-driven identification of shared mechanisms with co-occurring diseases through the investigation of shared disease-associated genes and pathways. To this end, we generated a comprehensive disease-gene map by combining disease-gene maps from different resources and using a semantic-derived knowledge-base [26] to map all those maps into *entrezgene*-ICD9 associations; *entrezgene* denotes the gene names from the NCBI database [27]. We assume genes that are mapped to two different diseases can be used as a proxy of the existence of common mechanisms between the two diseases [28, 29]. Hence, we used disease-gene associations to define *mechanistically derived* disease-disease associations.

The definition of these distances allowed us (i) to group and rank COPD-associated co-morbidities based on co-occurrence and/or mechanistic distance measurements; and, (ii) to identify candidate biomarkers that measure COPD-comorbidity status. Moreover, we characterize the novel candidate biomarkers linked to COPD co-morbidities by investigating their association with COPD risk factors such as smoking or physical inactivity. Finally, we compared the data-driven results with the state-of-the-art in the field and reported clinical knowledge.

## Methods

### Estimation of ϕ and Relative Risk (RR)

Generally, co-morbidity refers to the tendency of two diseases to appear in the same patient more frequently than expected by chance. Large-scale medical records allow for the systematic identification of such disease pairs. Here, we use the Medicare claims database introduced in [25, 30]. The diseases are ICD9-CM coded, we use the 3-digit level. Following previous work [25, 30–32], we use two complementary quantities to quantify the strength of the co-morbidity of two diseases *i* and *j*:

1. The Relative Risk, corresponding to the number of patients diagnosed with both diseases compared to the random expectation based on their prevalence in the general population:
  $$ R{R}_{ij}={C}_{ij}/\left({I}_i{I}_j/N\right), $$
  where *C*_*ij*_ is the number of patients affected by both disease and *I*_*i*_ and *I*_*j*_ denote the incidences of diseases *i* and *j* in a population of size *N*.
2. The Φ –correlation, which gives the Pearson correlation for binary variables:
  $$ {\varPhi}_{ij}=\left[\left(N\ {C}_{ij}\right)\ \hbox{--}\ {I}_i{I}_j\right]/\mathrm{sqrt}\left({I}_i{I}_j\left(N-{I}_i\right)\ \left(N-{I}_j\right)\right). $$

Using these definitions, we identify co-morbid diseases with a disease pair *i* and *j* for which *RR*_*ij*_ > 1 and Φ_*ij*_ > 0. Note that the two co-morbidity measures are not completely independent of each other and both have certain biases: For instance, Φ may provide small values even for highly associated diseases if their prevalences are very different, while *RR* may show abnormally large values for diseases with very small prevalence. Since the two measures are complementary in their respective biases, we consider both during the identification of co-morbidity associated pathways and biomarkers.

### Hierarchical clustering of COPD-associated diseases

To cluster the ICD9 COPD associated codes we computed a proximity measure between diseases as the inverse of the RR for all pairs of ICD9 codes. The hierarchical clustering was computed by using the hclust function in R [33]. The order of the diseases was retrieved and was given to the clinicians as the input for grouping the disease considering both the ordering and a clinical expertise.

### Gene disease map

The gene-disease map uses the following type of resources.

1. Gene-disease maps including CTD [34], PheGenI [35] and OMIM [36]. Additionally we include text-mining based mapping that we generated as part of the NCI cancer gene index [37] as well as further COPD specific text mining [38].
2. Disease ontologies including MeSH [39], ICD9 [40], ICD10 [41], the NCI Thesaurus [42] and SNOMED-CT [43].

For each integrated association we retained reference and evidence information as far as available in the original resource. Also this information can be used for subsequent filtering and ranking we decided to include all available associations for the integration step. We then used the UMLS Metathesaurus [44] to derive mappings between the different medical vocabularies and integrate the different gene-disease association resources which had used different disease vocabularies. The set of gene-disease associations used for analysis therefore represents the non-redundant sum of all individual integrated sources. All mappings and resulting gene – disease associations are publicly available in the COPD Knowledge-Base [26]. All those resources are detailed in the Additional file 1: Table S8. Most resources are publicly available.

### PCA analysis

Briefly, Principal Component Analysis (PCA) is a multivariate analysis method that identifies the components that maximally explains the variance of a given data-set. The first component is the vector explaining most of the variance; n component is the vector that explains most of the variance and is orthogonal to components n-1 to 1. PCA were computed in R using *PCA* function in the FactoMineR package [45].

### Ranking disease groups

Given a set of distance measurements between a disease group (DG) and COPD (SetM) the order of relevance of DGs was computed by ranking the DG using as a reference value the sum of all dist(Measure) where dist is the ranking of the DG using distance Measure for all Measures in SetM. For instance, the final ordering of DG is based in the sum of the rankings provided by Φ, RR, summarized gene-based distance (using JC and phi2) and summarized pathway-based distance (using the gene-sets of GO, Reactome and KEGG).

### Ranking genes and pathways

Genes were ranked based on their association to COPD-comorbidity. To this end first a matrix *mapping*1_*DG* was computed between DG and genes where 1 denotes gene-DG association and 0 otherwise. Then we computed a gene relevance measure using Φ (RR) as the ranking obtained from computing:

$$ relevance(geneX)={\displaystyle {\sum}_{DG} mapping1\hbox{--} D{G}_{geneX,DG}{\Phi}_{DG}.} $$

Relevance computes the sum of the Φ (similarly for RR) for those DGs that geneX has been associated to. The final ranking of a gene is based on the average of the ranks computed by using Φ and RR. The ranking was computed for both *mapping*1_*DG* and *mapping*2_*DG.*

In the case of pathways and gene-sets the measure is similarly computed but using the computed disease-gene_set matrices.

### Rank combination

**Fig. 1:**
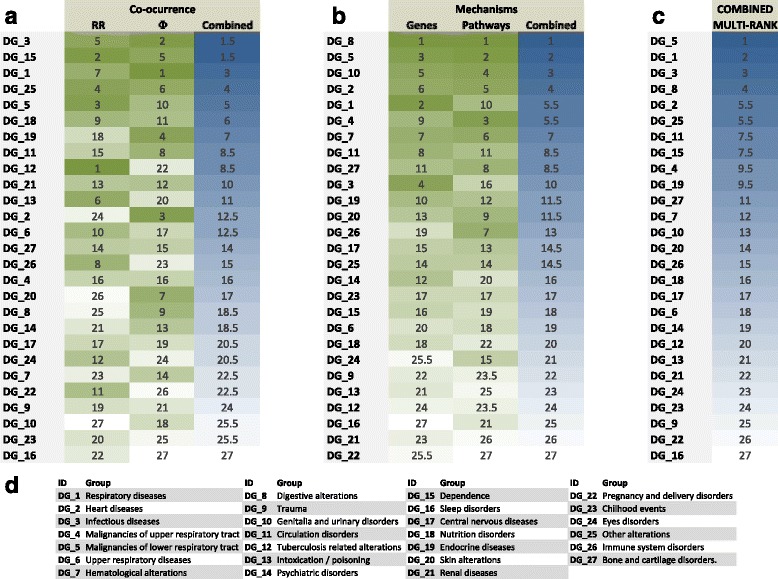
Disease groups and their association to COPD (ICD9 code = 496). Each row denotes a Disease Group identified (described in panel (**d**)). RR, Φ, Genes and Pathways denote the ranks of the distances between COPD and the DG; Combined columns denote the combined rank of (panel (**a**)) RR and Φ and (panel (**b**)) Genes and Pathways respectively. In panel (**c**), COMBINED denotes the final rank of the DG when all four ranks (RR, Φ, Genes and Pathways) are combined. In COMBINED DG5 (*malignancies in the lower respiratory track*) is the higher ranked disease group and DG16 is the lowest ranked (*parasomnias*). See Methods: *Rank combination* for details of how ranks are computed

Rank combination in disease groups is calculated by first *summing* for each DGs the individual ranks obtained from two measures (e.g. from RR and Φ); secondly the *sum* was used to rank again the disease groups. Similarly, to what is used in many ranking procedures, when multiple DGs obtain the same *sum* receive shared combined rank calculated from summing all occupied combined ranks and dividing by number of affected DGs. As an example from Fig. 1, DG1 has a Rank of 7 based on RR and a Rank of 1 based on Φ. The sum of both ranks is 8; when compared with the rest of sums from all DGs the new Rank of DG_1 is 3.

### Significance computed for genes and pathways

To estimate the Family Wise Error Rate (FWER) of a given gene based on the ranking we generated 10000 rankings as the sum of two random rankings considering the same total number of genes; for each permutation we computed the maximum value observed (maxperm). For each gene we estimated the FWER as “*number of times the rank observed was larger than maxperm*” divided by the total number of permutations.

### Overrepresentation analysis in gene-sets

For Gene Set Analysis we used the Reactome, KEGG and BioCarta gene sets contained in the Molecular Signature Database, MSigDB [46] and the Biological Processes category from Gene Ontology [47] we filtered for gene-sets with at least 20 genes and less than 200 in order to exclude too generic or too specific terms. To compute the enrichment of a disease-associated set of genes with a gene set we run the Fisher test [48]; Benjamini-Hochberg was used to adjust for multiple-testing [49]. A disease-associated set of genes was significantly associated to a gene-set if adjusted *p*-value < 0.1.

### Text-mining

We made use of Polysearch [50] and the novel PolySearch 2 [51] to search for associations between set of biomedical terms and genes. We used the basic settings except for the number of publications to be considered. Three sets of words were used: set1 = (“aging”, “age”), set2 = (“smoking”,”smoke”,”smoker”), set3 = (“training”,”healthy life style”).

## Results and Discussion

### Co-occurrence based on COPD co-morbidity analysis

#### Disease group associations with COPD

To identify COPD-associated diseases, we computed both Relative Risk (*RR*) and Pearson’s correlation for binary variables (Φ) between 3-digit ICD9-code diseases (ICD9 from now on) available in the health records of U.S. Medicare (Hidalgo et al, [25]). The total amount of patient records in Medicare was *N* = 13,039,018; all individuals are over 65 years, mostly white patients (>90 %) and there is an overrepresentation of females (58.3 %) [25]. For an initial assessment of COPD (ICD9 code 496) associated diseases, we selected all ICD9 codes with RR > 1.2. The set was named *ICD9selected*.

Many ICD9 codes have a very similar definition and it poses problems when doing analysis at 3-digit ICD9 level [18, 52, 53] because closely defined ICD9 codes show high co-morbidity between them. Aggregating ICD9 codes into groups have been applied before [54] however direct application of clustering algorithms is not optimal (see Additional file 2: Figure S1). In order to identify relevant sets of ICD9 codes with a shared clinical meaning we aggregated ICD9-codes into disease groups (DGs) using a 2-step process. First, we computed a hierarchical clustering of *ICD9selected* using *RR* as distance between codes (see the ordering in Additional file 3: Table S1). Second, combining the computational ordering of the hierarchical clustering with clinical expert knowledge, we grouped the ICD9-codes into DGs, as depicted in Additional file 3: Table S1, Additional file 4: Table S2 and Fig. 1d. Finally, the *RR* and Φ values between each DG and COPD were computed by considering that any individual was associated to a DG if the individual was diagnosed with at least one ICD9 code pertaining to the DG (see Methods).

As expected [25, 55, 56] we observed that *RR* and Φ often correlate only weakly. Therefore, in order to provide a global view (see Fig. 1a), we ranked DG-COPD associations using three measurements *RR*, Φ and their combined rank (see Methods). The top ranked DGs are Dependence (DG15) and Infectious diseases (DG3). We also observe expected disease groups such as Respiratory diseases (DG1) and Malignancies of lower respiratory track (DG5) in third and fifth rank respectively.

#### Changes over age of disease risk association

**Fig. 2:**
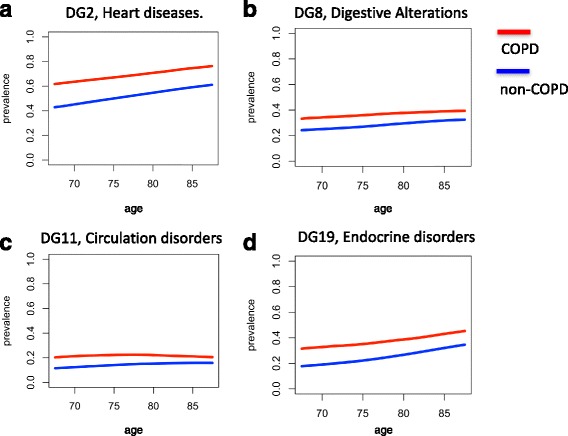
Prevalence of selected DG over age for COPD and non-COPD individuals. Each panel shows, for a given DG, the prevalence of the DG in non-COPD (*blue*) and COPD (*red*) individuals over windows of 5-years (e.g. 75 denotes the prevalence between 73 and 77 years both included). Prevalence is provided as a value between 0 and 1. DGs 2, 8, 11 and 19 are shown in panels (**a**), (**b**), (**c**) and (**d**) respectively. In all cases the prevalence is (as expected based on the selection of *ICD9selected*) higher in COPD individuals; however the differences between age are different among groups

We hypothesized that co-morbidity patterns of COPD could vary with age and/or gender. We therefore compared the prevalence of DGs in COPD and in non-COPD patients over age and gender using 5-year age windows (see Fig. 2). We identified two major types of age-associated co-morbidity progressions. In the first type there was a constant difference between prevalence in COPD and non-COPD while observing a growth of DG prevalence in both groups (Fig. 2a, b, d). In the second type, the differences in prevalence decreased with increasing age (Fig. 2c). Only in DG10 (*Genitalia and urinary disorders*) the difference in prevalence increased with age (see Additional file 5: Figure S2).

Additionally, we conducted a bootstrapping-based estimation of confidence intervals of the values observed; in most cases the confidence intervals were very small.

Additionally, we compared the prevalence of DG separately in men and women in COPD (summarized in Additional file 4: Table S2). When considering RR, co-morbidity is higher in females than in males for most DGs; especially in *Tuberculosis related alterations* (DG12), *Substance abuse related alterations* (DG15) and *Other alterations* (DG25). When considering Φ, we identified *Other alterations* (DG25) and *Bone and cartilage disorders* (DG27) more strongly associated in women; while *Respiratory diseases* (DG1), *Infectious diseases* (DG3), *Malignancies of lower respiratory tract* (DG5), *Digestive alterations* (DG8), *Circulation disorders* (DG11), *Nutrition disorders* (DG18), *Endocrine diseases* (DG19) and *Renal diseases* (DG21) are more strongly associated in men. We conclude that gender is a relevant co-morbidity covariate.

### Shared mechanisms in COPD co-morbidity

In our working hypothesis co-morbidity is the outcome of shared dis-regulated molecular mechanisms between DGs and COPD. Therefore, disease co-occurrence has a mechanistic component that, when uncovered, will provide insights into COPD. To identify comorbidity disease mechanisms, we use information about gene-disease associations. We first generated a comprehensive disease-gene map and then used it to compute *mechanistic-derived* association measures between diseases [28].

#### Integration-based disease-gene maps

**Fig. 3:**
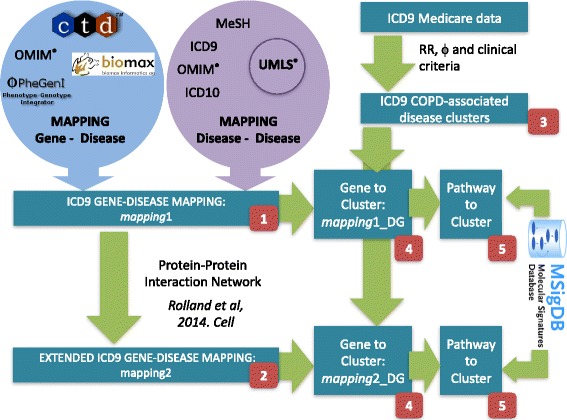
Framework to uncover co-morbidity associated mechanisms. The figure depicts first the use of gene-disease associations from multiple disease ontologies/nomenclatures and multiple disease-gene databases (and mappings from UMLS) to generate mapping1 (red block: 1) and its extension through PPI associations to generate mapping2 (red block: 2). Secondly, the figure depicts the computation of Disease Groups (red block: 3). Finally, the figure depicts the mapping of gene-DGs that results in mapping1_DG and mapping2_DG (red block: 4) and their pathway extensions (red block: 5)

In order to generate a comprehensive disease-gene map between ICD9 and *entrezgene* gene nomenclature, we first considered several disease-to-disease mappings (bridging between ontologies such as those in UMLS) and several disease-gene mappings (see Fig. 3); secondly, we integrated them into a COPD Knowledge-Base [26] including a semantic representation that allowed us to identify all associations between *entrezgene* genes and 3-digit ICD9 codes (*mapping*1). Figure 3 depicts the databases used during the mapping which was then used to generate a map between DG and *entrez* genes (*mapping*1_*DG*) by considering a gene-DG association if any of the ICD9 codes in a DG was associated to the gene.

Next we generated a map between ICD9 codes and gene sets. For each pair of gene-set and ICD9 code we used *mapping*1 to compute Fisher tests and then applied Benjamini-Hochberg [49] correction for multiple-testing (adjusted p-value). We included associations with an adjusted *p*-value < 0.01. The same procedure was applied for DG by using *mapping*1_DG. We considered the gene sets available in including KEGG, Reactome and Biocarta as derived from MSsigDB [46] and the Biological Processes category from GO [47, 57].

For many ICD9 codes and DGs only few associated genes were identified in *mapping*1 or *mapping*1_DG respectively, thus limiting the outcomes of the overrepresentation analysis (see Methods). To extend the mapping, we made use of the state-of-the-art Protein-Protein interaction network [58] that includes 14000 high-quality binary protein-protein interactions. We associated to each ICD9 (*DG*) those genes connected in the PPI to those connected to the disease (disease group) in mapping1 (*mapping1*_*DG*). We denote the new mapping *mapping2* (*mapping2*_*DG*). Using *mapping2* (*mapping2_DG*) a new PPI-derived gene-set vs ICD9 (*DG*) map were computed.

#### ICD9 and disease group (DG) distances to COPD

To define mechanistically derived disease-COPD distances we combined several complementary measures of association between COPD and disease groups using several layers of information: based on genes, based on pathways and based on their possible extensions by PPI. However, we investigated how to combine the information in a way that (1) optimizes heterogeneous sources while (2) excluding outliers.

When considering gene-disease and pathway-disease based associations between DGs and COPD we computed three different measures: (1) number of common features (*T*), (2) ratio between number of common features and the total number of pooled DG-COPD features (*Jaccard-type measurement, JC*), and (3) Pearson’s binary correlation (named *phi* in order to differentiate from disease co-occurrence Φ). Note that features may be refer to gene or pathways depending on the mapping used.

The methodology used to compute the final distances is described in the Additional file 6 (see also Additional file 7: Figure S3, Additional file 8: Figure S4, Additional file 9: Figure S5 and Additional file 10: Figure S6, Additional file 11: Figure S7 and Additional file 12: Figure S8 and Additional file 13: Figure S9). Briefly, we performed iterative distance selection where very similar distances were combined (GO, KEGG and REACTOME derived measures) and outliers were excluded (Biocarta-based distances). Finally, two disease-COPD measures were considered: gene-based and gene-set-based distances (see Methods); see Additional file 6), each one combining the three measures *T*, *JC* and *phi*. The mechanistically derived ranking is summarized in Fig. 1b.

We combined the ranking-based distances of genes and gene-sets (Fig. 1b) and co-ocurrence based measurements (Fig. 1a) into a single final measure by ranking over the sum of all individual distances (see Fig. 1c). As expected, we observed among the top-ranked DGs: DG5 (*Malignancies in the lower respiratory tracks*) and DG1 (*Respiratory disease*s). Furthermore, among the top ones, we identified known COPD-associated disease groups such as *Heart Diseases* (DG2). Interestingly, we identified a novel disease group as first ranked: *Digestive Alterations* (DG8).

Next, we performed a manual exploration of our distance calculations by manually examining the strongest co-morbidity and mechanistic association, COPD – DG5 to see whether the association reflects biomedical expertise. To this end, we identified which features are shared between COPD, DG5 and each of the ICD9 codes in DG5 (Additional file 7: Figure S3); when considering gene-sets KEGGs *Focal Adhesion*, *Renal Cell Carcinoma* and *Melanoma* and GO-Biological Processes *Positive Regulation of Cell Proliferation*, *Behaviour*, *Regulation of Protein Metabolic Process* and *Chemical Homeostasis* are shared; many cancer related and/or generic pathways are also observed. When investigating the genes we identified several associated with the MAP-Kinase pathway (e.g. BRAF, MAP3K8) and the immune system (e.g. IL1, IL1R, and TNFRSF11B) (Additional file 7: Figure S3); importantly, the MAP-Kinase pathway has been associated with both COPD ([59–61] and malignancies in the lower respiratory tracks. We acknowledge that the set of genes and pathways identified may show a bias towards the large amount of positive results gathered about cancer in databases; however, we tried to minimize such effect by using the PPI-based extension association that is generated based on unbiased high-throughput experimental evidence [58].

#### Principal candidate markers of disease co-morbidity: genes and pathways

**Fig. 4:**
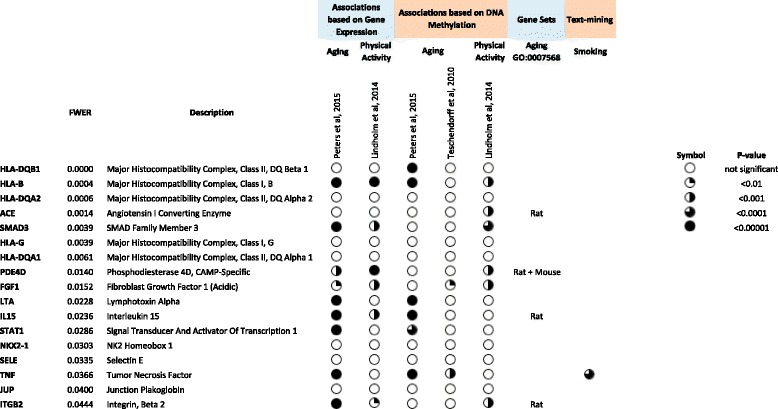
Candidate biomarkers for COPD-comorbidity. Included are the genes selected as candidate biomarkers for FWER < 0.05 (see Methods). For each gene it is shown if it has been associated to Smoking, Aging and/or Physical activity based on gene expression (see Additional file 16: Table S7), DNA Methylation (see Additional file 16: Table S7) or PolySearch-derived [50, 51] text-mining analysis (see Additional file 15: Tables S4, S5 and S6)

By combining co-occurrence information (*RR* and Φ) with disease-gene mappings we aimed to identify the most relevant genes and pathways associated with COPD co-morbidity. For each gene we computed a score that sums the RR values of each of the DGs the gene is associated with. The value is then used to rank all genes. Similarly, scores and rankings are computed using Φ; and lastly a final ranking is computed by combining both Φ-derived and RR-derived rankings. The Family Wise Error Rate (FWER) was computed (see Methods) and genes with FWER < 0.05 are shown in Fig. 4.

Importantly, the top 3 genes are Human Leukocyte Antigen genes (HLA), which are major histocompatibility complexes: HLA-DQB1 (associates with diabetes mellitus among others), HLA-B (associated with immunodeficiency) and HLA-DQA2 (associated with diabetes mellitus and celiac disease). Also from the HLA family is significantly identified HLA-G (tumor scape) and HLA-DQA1 (associated to diabetes mellitus [62], celiac disease [63] and juvenile idiopathic arthritis [64] among others). Among the non-HLA markers we identify relevant markers such as ACE, Angiotensin I Converting Enzyme [65] which is associated to cardiovascular complications, and SMAD3 that mediates multiple signaling pathways and TGF-beta-mediated transcription [66]. In addition, we observe many immune-associated genes, such as STAT1 and IL15. We performed a similar analysis using gene-sets to identify top-candidate gene-sets associated to co-morbidity. Significant results are shown in Additional file 14: Table S3.

Finally, we investigated if the identified COPD-comorbidity associated genes have also been associated to smoking, aging or physical activity. We first used a text-mining approach (PolySearch [50] and PolySearch 2.0 [51], see Methods); the results of the queries are shown in Additional file 15: Tables S4, S5 and S6 respectively. Only TNF was found associated with smoking.

Secondly, and in order to overcome text-mining biases [67] we investigated the selected genes in High-throughput based studies of gene expression and/or DNA Methylation for smoking, age and training (as a proxy for physical activity); see description in Additional file 16: Table S7.

While we acknowledge that results of those studies (see Fig. 4) may depend in selection criteria, number of individuals and ethnicity among others, we obtain consistent observations: (a) no selected genes were found significant in smoking studies, (b) some selected genes have been associated to age and/or gender. Finally, SELE, TNF and JUP, which are not associated with age, gender or smoking are relevant candidates to be considered in further studies; associations of these three genes with DGs are reported in Additional file 1: Table S8.

## Conclusions

We show that by integrating co-occurrence information with gene-disease mappings it is possible to rank disease co-morbidities and to identify co-morbidity features of interest (such as genes and pathways) and possibly help uncover the underlying disease mechanisms. In this process we have generated no original raw data but we have made use of existing repositories and knowledge-bases available.

In this work, we first grouped COPD-associated ICD9 codes into clinically relevant disease groups which were then ranked based on co-occurrence measures (RR and Φ). By this approach we identified in top associated expected diseases such as *Respiratory Diseases* and *Malignancies in the Lower respiratory track* both considered as positive controls; we also identified the genes involved in the association such as BRAF and IL1. Furthermore, we showed that COPD co-morbidity depends on age and gender, with different patterns of dependency for different diseases. We consider that this observation warrants more detailed future studies aimed at updating clinical management and diagnosis of COPD such as GOLD clinical protocols. Interestingly, only in DG10 (*Genitalia and urinary disorders*) the difference in prevalence increased with age (see Additional file 5: Figure S2). This observation is supported by [68] and we hypothesize that it can explained by age-related muscle dysfunction in elderly patients with COPD [12, 13], which may affect urinary muscles.

**Fig. 5:**
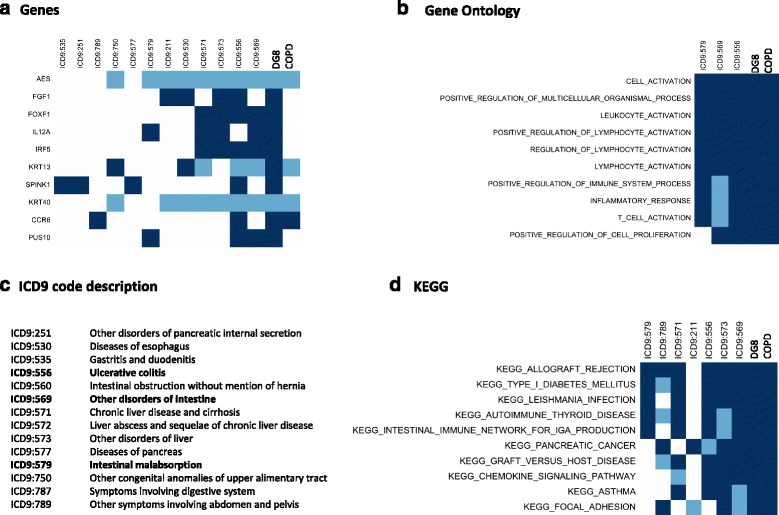
Genes and Pathways relating COPD and Digestive Alterations Disease Group (DG8). The figure shows the association between genes (**a**), Gene Ontology (**b**) and KEGG (**d**) gene-sets for those ICD9 codes included in DG8; the description of ICD9 codes is provided in panel (**c**). A dark (light) blue square denotes if the association between disease and pathway or gene was computed as significant when using either *mapping*1_*DG* or *mapping*2_*DG* (only *mapping*1_*DG*). Top 10 genes or gene-sets are shown; and then only ICD9 codes with at least association with an item are shown. The ICD9 codes are ordered using the number of associations with genes or gene-sets in the total set; from lower (left) to higher (right). The last two elements denote the association with DG8 and COPD. Similar information for Biocarta and Reactome is depicted in Additional file 12: Figure S8. In panel (**c**), those ICD9 codes shown in all other panels are in bold

We combined a robust mechanistic-derived ranking of COPD-comorbidity (based in shared genes or pathways, Fig. 1b) with a co-ocurrence derived ranking (Fig. 1a). The identified ranking (Fig. 1c) contains sufficient positive controls to also investigate the novel top-associations in more detail. For instance among those DG5, *Malignancies of lower respiratory track*, includes lung cancer which is one of the top 3 causes of death in patients with COPD [7, 69]. The most interesting novel association was DG8, *Digestive disorders*. There are studies supporting this association starting from 1991 when it was observed a significant co-occurrence between COPD and oesophageal-gastric and duodenal disease [70]. More recently, it has been shown that COPD patients were more likely to consult about *digestive system diseases* (Odd Ratio: 1.31; 95 % CI 1.02-1.68) [71]. Keely S et al, [72] proposed that ischemia-driven loss of epithelial barrier function may represent an underlying cause and chronic nature of many gastro-intestinal diseases in patients with COPD. Of all the 32 ICD9 codes contained in DG8, only a subset were associated to genes and/or pathways; out of these, the strongest associated codes were: *Other disorders of intestine*, *Ulcerative colitis* and *Intestinal malabsorption*. Importantly, the co-morbidities between COPD and ulcerative colitis (Ekbom et al, [73]) and chronic liver disease [74] respectively, have been reported previously, as well as, generally, the co-morbidity between COPD and digestive alterations [71, 75]. Interestingly, the top KEGG pathways linking DG8 and COPD are associated to third diseases such as Type 1 Diabetes, Asthma and Pancreatic Cancer (Fig. 5d), but also to specific pathways, such as the Intestinal Immune Network for IgA production (Fig. 5d) (which describes the production of non-inflammatory immunoglobulin A antibodies that serve as defense against micro-organisms) and other immune associated pathways. Among the top genes shared between COPD and DG8, we identified: AES (NF-kappa-B-regulated gene expression), KRT13 (Keratin 13, Type1), KRT40 (Keratin 40, Type1) and CCR6 (Chemokine C-C Motif Receptor 6; relevant in antigen-driven B-cell differentiation).

When comparing the analysis outcomes of top-ranked DGs with known COPD co-morbidities (such as Type 2 Diabetes (T2D), Cardiovascular Diseases [76, 77] we found all expected diseases but DT2. We computed the associations between COPD (ICD9 code 496) and ICD9 code 250 (Diabetes) by *RR* (1.027) and Φ (0.005). We acknowledge that by this data-driven approach Diabetes is not being selected based on a threshold selection; however, many pathways associated to T2D and metabolic-associated diseases are being selected by our methodology. Furthermore, we made 5 groups of ICD9 codes for anxiety, depression, diabetes, heart failure and ischemic heart respectively (classical COPD-comorbidities); for all group we also computed the age-window prevalence plots (Additional file 8: Figure S4). We observe that for anxiety and depression there is a difference in prevalence in COPD and non-COPD patients that decreases for elderly individuals. The difference is large, and remains constant, for heart failure and ischemic heart while it is small and constant for diabetes.

When investigating top COPD-comorbidity markers, we ranked genes based on their association with COPD-comorbidity by combining disease-gene information and co-occurrence measurements (Φ and RR). We again consider the top identified genes as positive controls, as most of them are Human Leukocyte Antigen (HLA) genes; most of these genes are shown associated from GWAS and genotype studies in immune associated diseases [78, 79] and cancer [80]; furthermore it has been shown that in many diseases patients need to be stratified based on HLA genotypes, such as in [81]. Despite that the identification of HLA-genes in the top of the list may reflect real co-morbidity associations and/or a bias towards deeply-genotyped diseases; we consider that further studies are required to address this question, but in any case HLA-genes are arguably major candidates for co-morbidity status. Among the non-HLA markers IL15 has already been associated to the chronic cavitary pulmonary aspergillosis [82] and to virus-induced COPD exacerbations [83] and, importantly, IL15 has been associated with COPD severity [84]. We consider IL15 as a relevant biomarker candidate for addressing COPD comorbidity status however IL15 has also been associated to aging and physical activity.

From the selected candidate biomarkers there are three genes that have not been associated to any other confounders (such as age, gender and training): Selectin E (SELE, part of the selectin family of cell adhesion protein; found in cytokine stimulated endothelial cells), Tumor Necrosis Factor (TNF, necessary in the induction of acute response, which includes the production of C-Reactive Protein; produced by several immune cells) and Junction Plakoglobin (JUP, part of catenin family and encodes major cytoplasmic protein). Only TNF have been previously associated to COPD co-morbidity [85, 86]. This result does not exclude possible associations between candidate genes (SELE, TNG or JUP) and shared risk factors however, in the search of co-morbidity biomarkers, our results in Fig. 4 prioritize them against other genes with risk factor associations.

We acknowledge that all reported results should be considered within the following limitations: (i) disease-gene associations are biased towards published (positive) results, (ii) ICD health records have biases and may differ from countries and (iii) some of the disease groups identified have a very broad definition that may affect their relevance such as DG23 and DG25. However, despite possible limitations and biases of our “*data and mapping driven*” methodology we are able to highlight the need to include digestive alterations in future studies addressing COPD co-morbidity and an initial set of candidates that drive such association mechanistically. Furthermore, we identified a set of genes as candidate biomarkers for COPD co-morbidity.

## Additional files

Additional file 1: Table S8. Association between genes not-associated with age, smoking or life-style and disease groups. In each cell, a 2 (1) denotes that association between gene and disease cluster was identified using *mapping1_DG* and *mapping2_DG* (only when *mapping2_DG*). (XLSX 38 kb)

Additional file 2: Figure S1. Heatmap of ICD9 codes associated with COPD. RR-based heatmap between 3-digit ICD9 COPD associated disease codes (RR > 1.5). (a) Complete heatmap without reordering. The size and color of each square denotes the strength of the association in RR. The heatmap is showing the ICD codes ordered alphabetically. (b) Detail of a section of the heatmap with RR-based highly associated codes that show very similar definitions of codes. (PDF 3755 kb)

Additional file 3: Table S1. Disease groups in detail. ICD codes are shown as ordered by a RR-based hierarchical clustering; the tables includes the information of co-occurrence between COPD and the ICD9 codes selected in *ICD9selected*. The columns of the table denote: ICD9: the ICD9 code. GROUP: the DG the ICD9 code pertains to. NAME: the name of the ICD9 3-digit group. prevalence ICD: prevalence of ICD9 code in Medicare. common_diagnoses: prevalence of ICD9 code and COPD simultaneously. RR: COPD-ICD9 code relative risk. Φ: COPD-ICD9 code Φ. (XLSX 46 kb)

Additional file 4: Table S2. COPD and Disease Groups by gender. For each DGs the co-occurrence of a DG and COPD is studied by gender and also the differences between genders are computed. For each gender the following columns show: prevalence ICD: prevalence of ICD9 code in Medicare. common_diagnoses: prevalence of ICD9 code and COPD simultaneously. RR: COPD-ICD9 code relative risk. Φ: COPD-ICD9 code Φ. %: the proportion of COPD individuals that also are diagnosed with DGs. Additionally differences between Male and Female are computed for RR, Φ and %. And the ratio of the differences and the Male Values. (XLSX 30 kb)

Additional file 5: Figure S2. Prevalence of selected DG10 (Genitalia and urinary disorders) over age for COPD and non-COPD individuals. DG prevalence in non-COPD (blue) and COPD (red) individuals over windows of 5-years (e.g. the 75 age denotes the prevalence between 73 and 77 years both included). Prevalence is computed between 0 and 1. In this case the prevalence difference between populations increases over time. In (a) the prevalence is depicted between the maximum 1 and the minimum 0, while in (b) the prevalence is zoomed into the ranges of the DG10. (PDF 384 kb)

Additional file 6: Supplementary Materials and Methods. (DOCX 18 kb)

Additional file 7: Figure S3. Genes and Pathways relating COPD and Malignancies of Lower Respiratory Track (DG_5). The figure shows the association between genes (a) and (b, c) pathways for those ICD9 codes included in DG_5. A dark (light) blue square denotes that the association between disease and pathway or gene was computed as significant when using either *mapping*1_*DG* or *mapping*2_*DG* (only *mapping*1_*DG*). Selection criteria for the display of diseases, genes and gene-sets are the same as those described in Fig. 5. (PDF 1143 kb)

Additional file 8: Figure S4. Targeted Co-morbidity. Prevalence differences over age windows for targeted ICD9 codes in Medicare. DG prevalence in non-COPD (blue) and COPD (red) individuals over windows of 5-years (e.g. the 75 age denotes the prevalence between 73 and 77 years both included). Prevalence is computed between 0 and 1. For each disease in the left plot the prevalence is depicted between the maximum 1 and the minimum 0, while in the right plot the prevalence is zoomed into the ranges of the DG10. (PDF 1767 kb)

Additional file 9: Figure S5. Genes and Pathways relating COPD and DG8. The figure shows the association between Reactome (a) and Biocarta (b) pathways for most-associated ICD9 codes included in DG8. A dark (light) blue square denotes that the association between disease and pathway or gene was computed as significant when using either *mapping*1_*DG* or *mapping*2_*DG* (only *mapping*1_*DG*). The description of the ICD9 codes is provided in panel (c). Additional file 9: Figure S8 extends the information provided in Fig. 5 and follows the same color-code and selection criteria. (PDF 1550 kb)

Additional file 10: Figure S6. Ranked based distances between DG and COPD. Each column denotes the ranking of distances (from 1 to 27, larger is closer) between each DG and COPD. JC, T and PHI denote respectively Jaccard-type, Total and *phi* distance. Genes, KEGG, REAC, BIOC and GO denote respectively KEGG, Reactome, BioCarta and Gene Ontology gene sets. EXT denotes distance computed with extended gene-disease associations by PPI. Φ and RR denote the co-occurrence based distances. (PDF 293 kb)

Additional file 11: Figure S7. PCA from the data displayed in Additional file 7: Figure S6. Both panels are showing the same information with different color-coding to highlight specific results. (a) Color-code to show the different types of measurements: JC, T, *phi* or co-occurrence based measures. (b) Color-coded to show the different sources of information: genes, gene-sets and co-occurrence based measurements. (PDF 329 kb)

Additional file 12: Figure S8. Ranked based distances between DG and COPD from Step 2. Ranked based distances between DG and COPD. Each column denotes the ranking of distances (from 1 to 27, larger is closer) between each DG and COPD. JC, and PHI denote respectively Jaccard-type and *phi* distance. Genes, KEGG, REAC, BIOC and GO denotes respectively KEGG, Reactome, BioCarta and Gene Ontology gene sets. Φ and RR denote the co-occurrence based distances. (PDF 165 kb)

Additional file 13: Figure S9. PCA from the data displayed in Additional file 9: Figure S8. Both panels are showing the same information with different color-coding to highlight specific results. (a) Color-code to show the different types of measurements: JC, phi or co-occurrence (Φ and RR) based measures. (b) Color-coded to show the different sources of information: genes, gene-sets and co-occurrence based measurements. (PDF 191 kb)

Additional file 14: Table S3. Pathway co-morbidity biomarkers for DG_8. (XLSX 9 kb)

Additional file 15: Tables S4, S5 and S6. Text-mining analysis by PolySearch. set1 = (“aging”, “age”), set2 = (“smoking”,”smoke”), set3 = (“training”,”train”,”healthy life style”); the results of the queries are shown in Additional file 14: Tables S4, S5 and S6 respectively. (ZIP 97 kb)

Additional file 16: Table S7. Data-sets selected to investigate genes in the context of aging, smoking and life-style. (XLSX 33 kb)
