# Supplementary figures and images for: From comorbidities of chronic obstructive pulmonary disease to identification of shared molecular mechanisms by data integration
Source: BMC Bioinformatics. 2016 Nov 22;17(Suppl 15):23–35. doi: 10.1186/s12859-016-1291-3 (PMC5133493; doi:10.1186/s12859-016-1291-3)

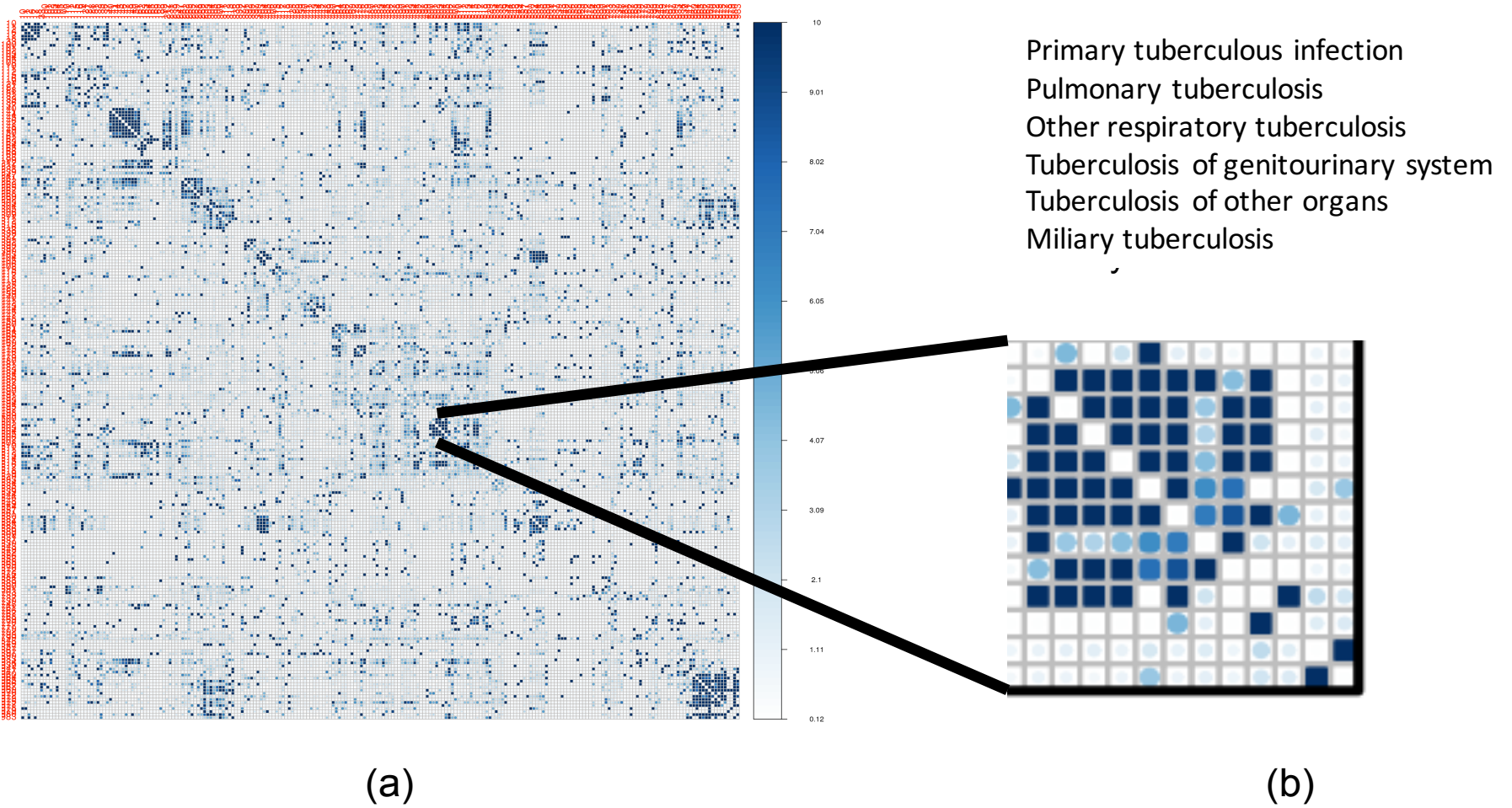

Fig. S1

Supplement: Supplementary file 2 — Heatmap of ICD9 codes associated with COPD. RR-based heatmap between 3-digit ICD9 COPD associated disease codes (RR > 1.5). (a) Complete heatmap without reordering. The size and color of each square denotes the strength of the association in RR. The heatmap is showing the ICD codes ordered alphabetically. (b) Detail of a section of the heatmap with RR-based highly associated codes that show very similar definitions of codes. (PDF 3755 kb) [file 12859_2016_1291_MOESM2_ESM.pdf]

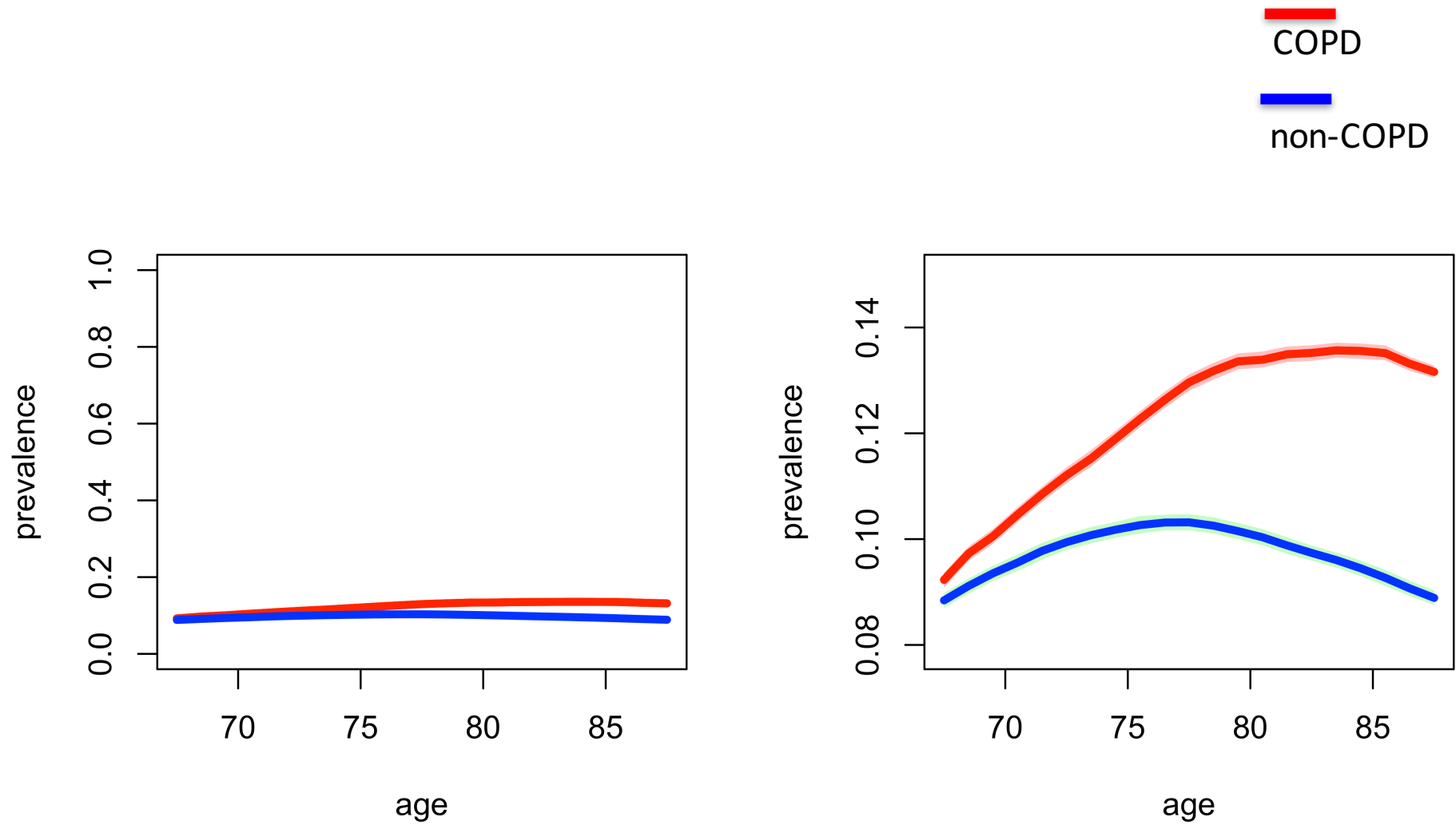

**Fig. S2**

Supplement: Supplementary file 5 — Prevalence of selected DG10 (Genitalia and urinary disorders) over age for COPD and non-COPD individuals. DG prevalence in non-COPD (blue) and COPD (red) individuals over windows of 5-years (e.g. the 75 age denotes the prevalence between 73 and 77 years both included). Prevalence is computed between 0 and 1. In this case the prevalence difference between populations increases over time. In (a) the prevalence is depicted between the maximum 1 and the minimum 0, while in (b) the prevalence is zoomed into the ranges of the DG10. (PDF 384 kb) [file 12859_2016_1291_MOESM5_ESM.pdf]

**(a) Anxiety**

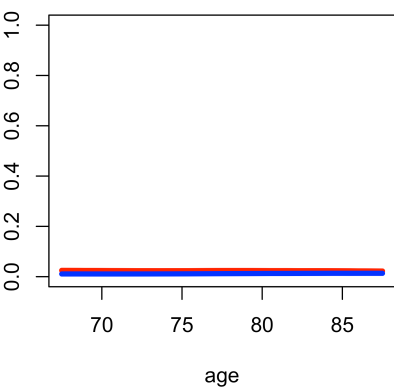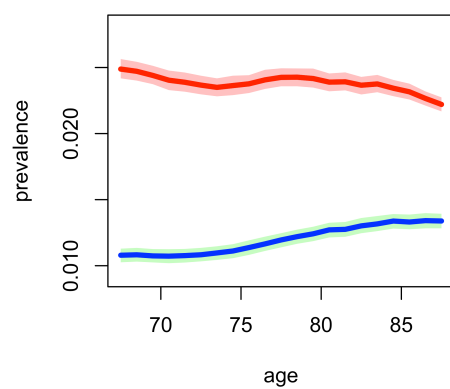

**(b) Depression**

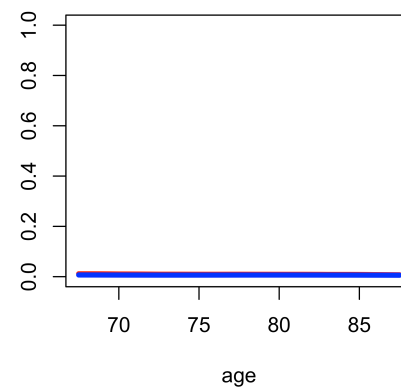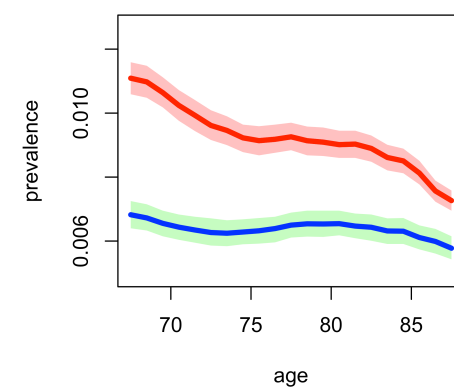

**(c) Diabetes**

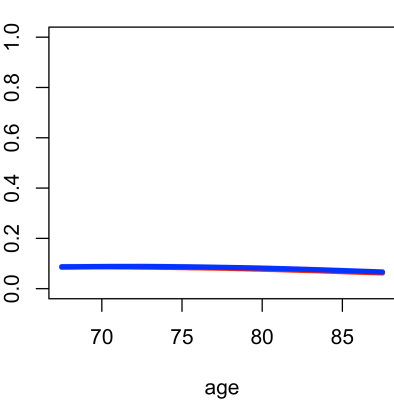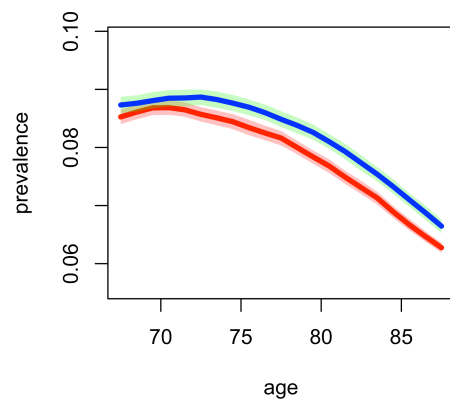

**(d) Heart failure**

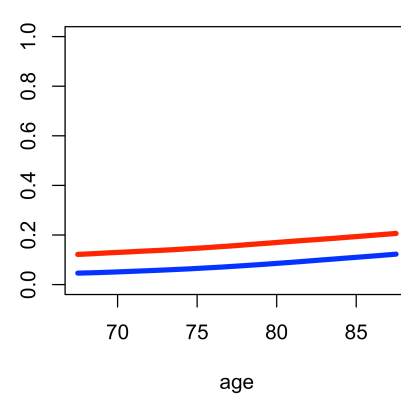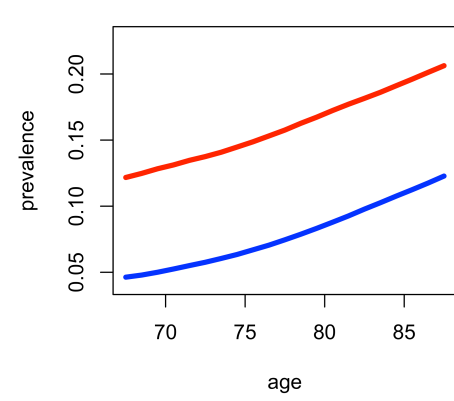

**(e) Ischemic heart**

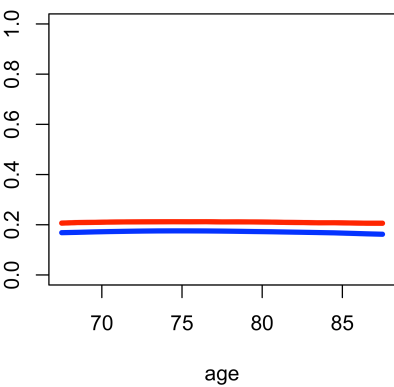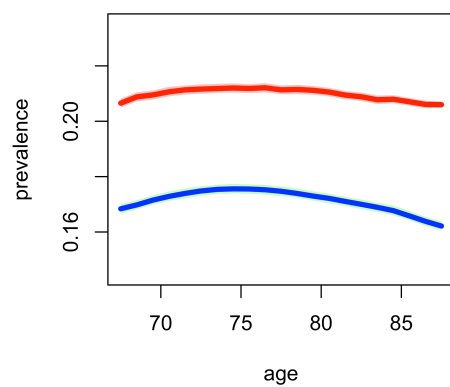

**Fig. S4**

Supplement: Supplementary file 8 — Targeted Co-morbidity. Prevalence differences over age windows for targeted ICD9 codes in Medicare. DG prevalence in non-COPD (blue) and COPD (red) individuals over windows of 5-years (e.g. the 75 age denotes the prevalence between 73 and 77 years both included). Prevalence is computed between 0 and 1. For each disease in the left plot the prevalence is depicted between the maximum 1 and the minimum 0, while in the right plot the prevalence is zoomed into the ranges of the DG10. (PDF 1767 kb) [file 12859_2016_1291_MOESM8_ESM.pdf]

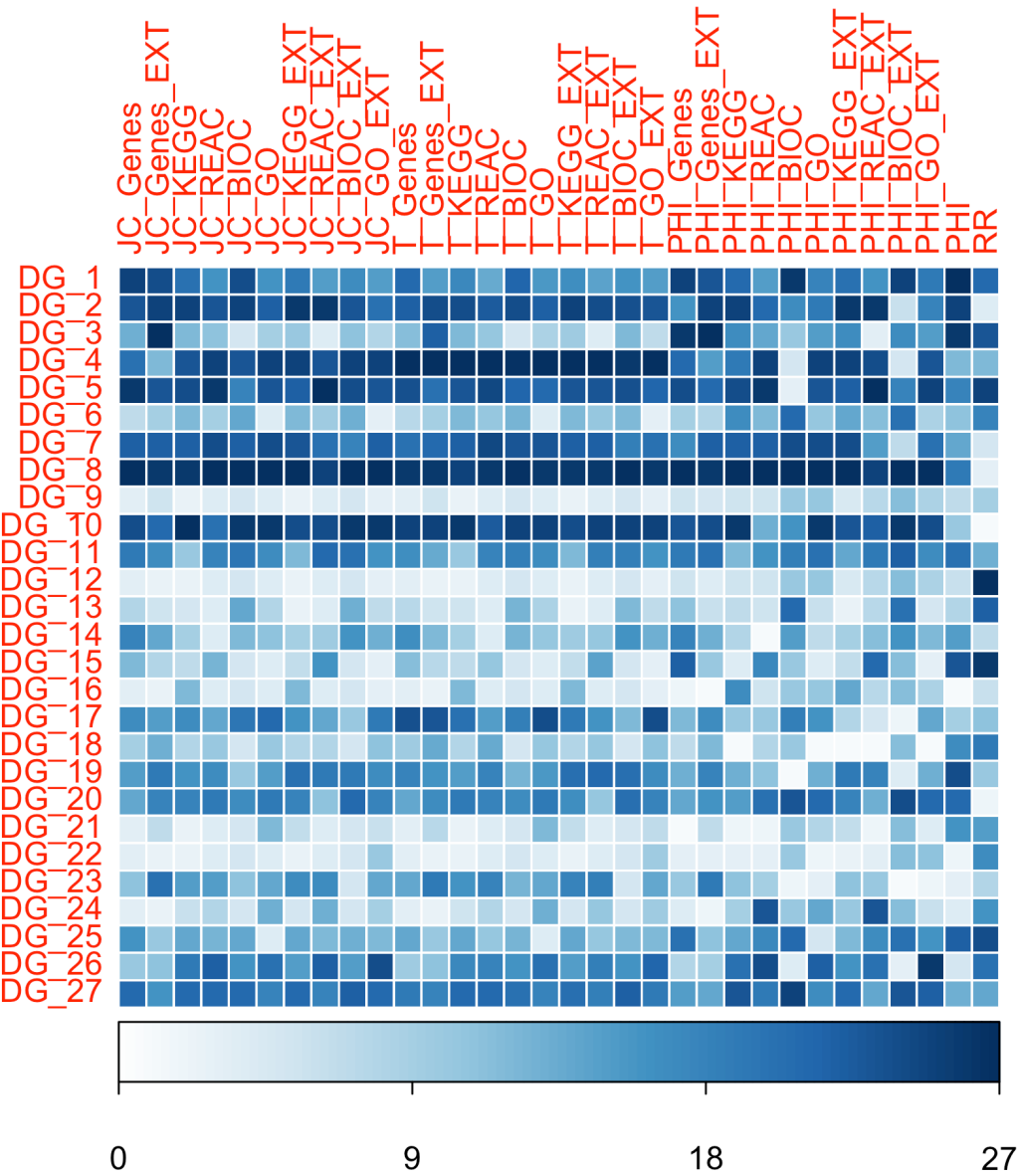

**Fig. S6**

Supplement: Supplementary file 10 — Ranked based distances between DG and COPD. Each column denotes the ranking of distances (from 1 to 27, larger is closer) between each DG and COPD. JC, T and PHI denote respectively Jaccard-type, Total and phi distance. Genes, KEGG, REAC, BIOC and GO denote respectively KEGG, Reactome, BioCarta and Gene Ontology gene sets. EXT denotes distance computed with extended gene-disease associations by PPI. Φ and RR denote the co-occurrence based distances. (PDF 293 kb) [file 12859_2016_1291_MOESM10_ESM.pdf]

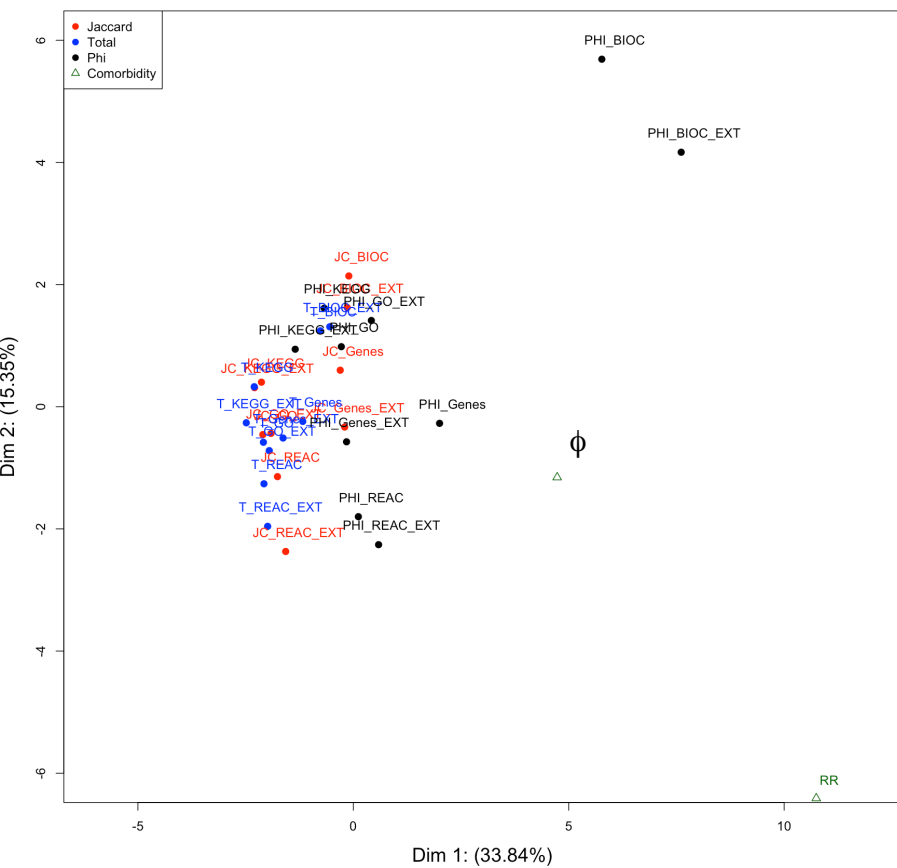

(a)

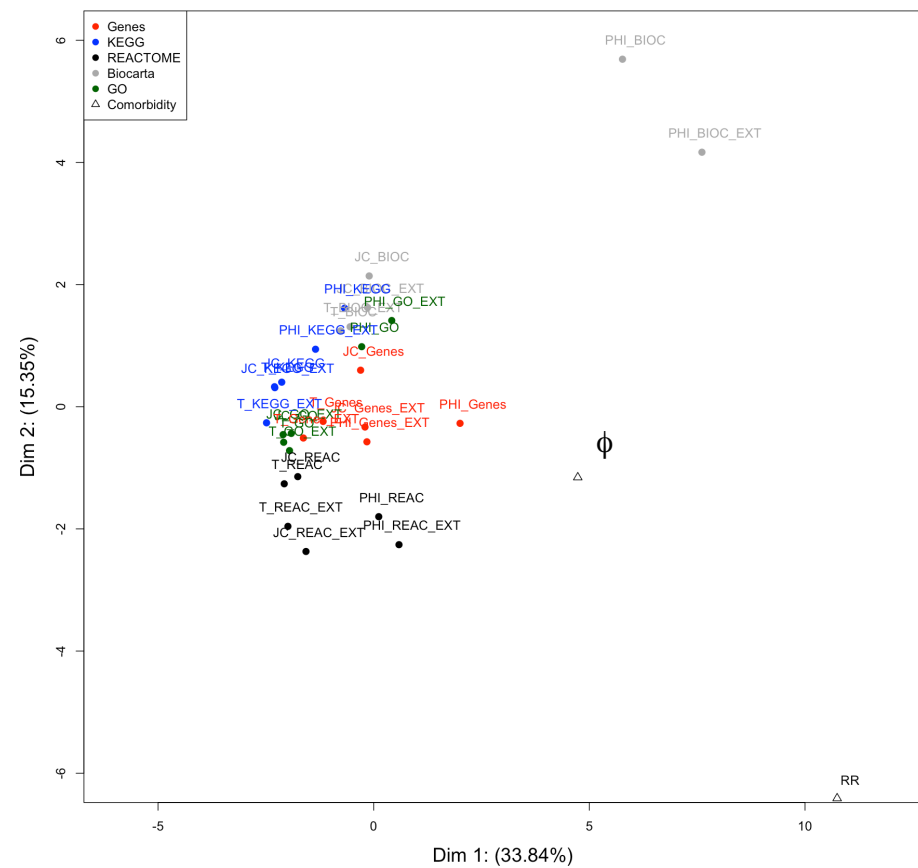

(b)

Fig. S7

Supplement: Supplementary file 11 — PCA from the data displayed in Additional file 7: Figure S6. Both panels are showing the same information with different color-coding to highlight specific results. (a) Color-code to show the different types of measurements: JC, T, phi or co-occurrence based measures. (b) Color-coded to show the different sources of information: genes, gene-sets and co-occurrence based measurements. (PDF 329 kb) [file 12859_2016_1291_MOESM11_ESM.pdf]

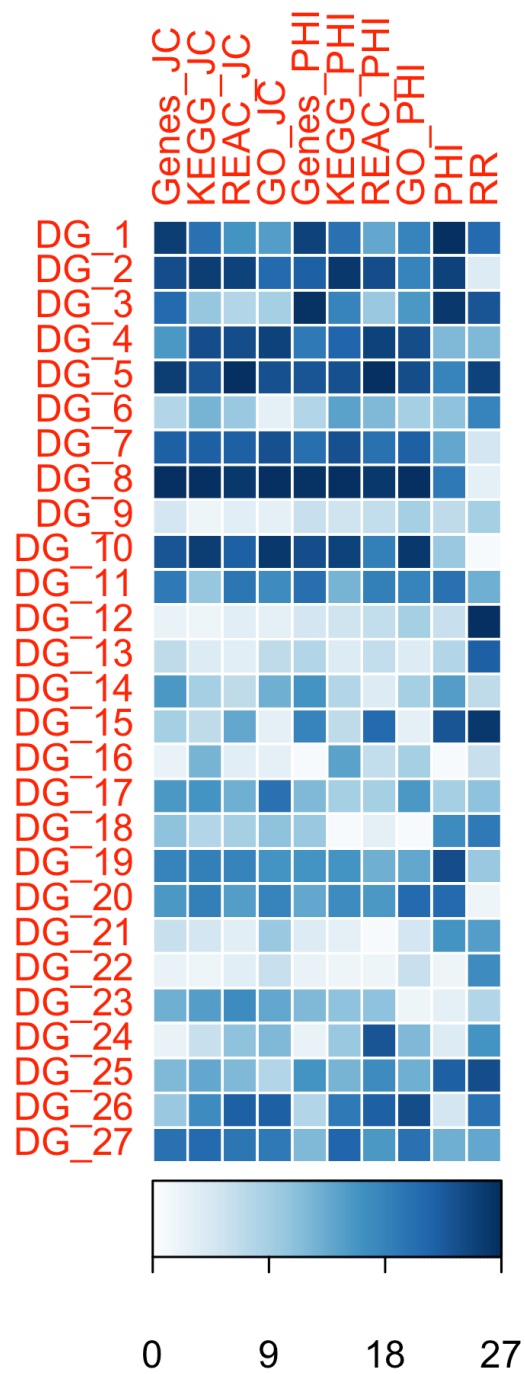

**Fig. S8**

Supplement: Supplementary file 12 — Ranked based distances between DG and COPD from Step 2. Ranked based distances between DG and COPD. Each column denotes the ranking of distances (from 1 to 27, larger is closer) between each DG and COPD. JC, and PHI denote respectively Jaccard-type and phi distance. Genes, KEGG, REAC, BIOC and GO denotes respectively KEGG, Reactome, BioCarta and Gene Ontology gene sets. Φ and RR denote the co-occurrence based distances. (PDF 165 kb) [file 12859_2016_1291_MOESM12_ESM.pdf]

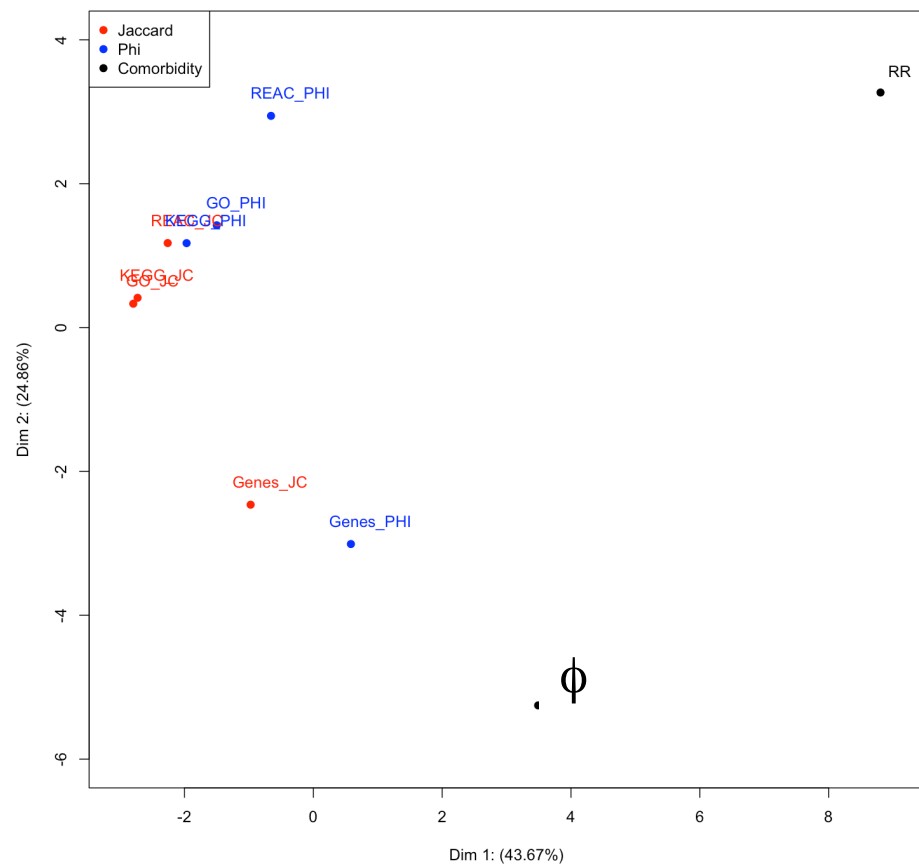

(a)

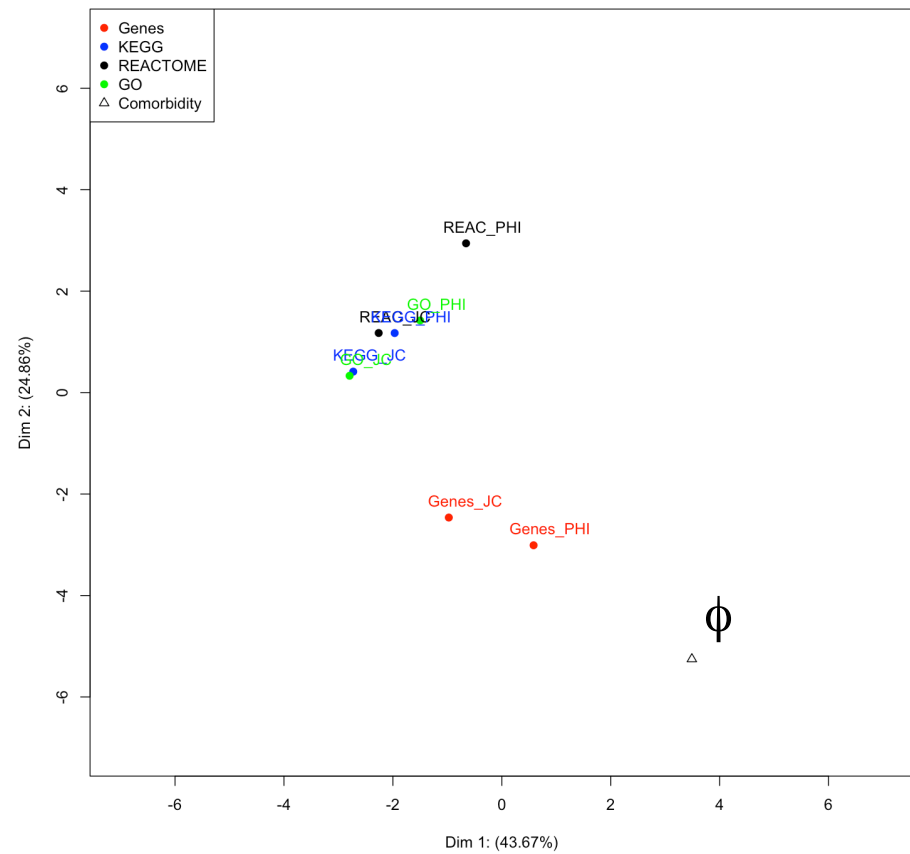

(b)

Fig. S9

Supplement: Supplementary file 13 — PCA from the data displayed in Additional file 9: Figure S8. Both panels are showing the same information with different color-coding to highlight specific results. (a) Color-code to show the different types of measurements: JC, phi or co-occurrence (Φ and RR) based measures. (b) Color-coded to show the different sources of information: genes, gene-sets and co-occurrence based measurements. (PDF 191 kb) [file 12859_2016_1291_MOESM13_ESM.pdf]
