# Supplementary material for: From comorbidities of chronic obstructive pulmonary disease to identification of shared molecular mechanisms by data integration
Source: BMC Bioinformatics. 2016 Nov 22;17(Suppl 15):23–35. doi: 10.1186/s12859-016-1291-3 (PMC5133493; doi:10.1186/s12859-016-1291-3)
Supplement: Supplementary file 6 — Supplementary Materials and Methods. (DOCX 18 kb) [file 12859_2016_1291_MOESM6_ESM.docx]

**Supplementary Methods.**

**Computing Disease-COPD distances based on genes and pathways.**

In several steps we discarded distances that were considered as outliers and merged those that were very similar; the underlying idea was to obtain a robust set of distances that combined co-occurrence, gene and pathway based distances.

Step 1: For each measurement we ranked the DG-COPD associations as shown in **Fig. S6**. For instance, DG_8 (DG12) appears high (low) in most measurements; when considering no co-occurrence measurements we observe high correlation between rankings. We observed that *RR* and Φ do not correlate in many cases (as expected [1–3]) but they also do not correlate with non-co-occurrence measurements. We did a Principal Component Analysis (PCA, **Fig. S7**) and observed the following: (1) the combined use of the first component (33.84% variance explained) and second component (15.35% variance explained) separates primarily based on the origin of the measurements (genes, gene-sets, etc.) and secondly the *phi* measurements from the JC and T measurements. (2) Additionally we observed that measurements using the regular disease-gene set or the extended by PPI disease-gene set always appeared close and showed high correlation. (3) Co-occurrence based measurements, *RR* and *phi,* are separated from the rest. And (4) Biocarta-based distances are outliers compared to the rest of the gene-sets. Based on those observations we decided to (i) exclude Biocarta distances (as an outlier) and (ii) exclude T measurements considering that JC and T were very similar, and (iii) compute an average rank adding the ranks provided by the original gene-disease set association and the extended by PPI associations.

Step 2: The analysis of the new distances is provided in **Fig. S8** (ranked-based distance) and their projections in two dimensions in **Fig. S9** (PCA). Interestingly the PCA show that using first (43.67%) and second (24.86%) components the new distances are separated into 4 types: gene-driven distances, gene-set driven distances and the two co-occurrence based distances. This is a very relevant result because it highlights that the three gene-set definitions (GO, KEGG and REACTOME) provide very similar distances between DG and COPD, confirming the robustness of the approach. Furthermore, JC and *phi* measurements are very similar.

Step 3: Based on this result we computed a ranked-based distance from the sum of the distances of the three gene-sets and a rank-based distance based on the sum of both JC and *phi* distances for gene and gene-set distances, see **Fig. 1(b)**.

**References**

1. Cohen J, Cohen P, West SG, Aiken LS. Applied Multiple Regression/Correlation Analysis for the Behavioral Sciences. Routledge; 2002.

2. Katz D, J. B, Azen SP, M.C. P. Obtaining Confidence Intervals for the Risk Ratio in Cohort Studies. Biometrics. 1978;34:469–74.

3. Hidalgo CA, Blumm N, Barabási A-L, Christakis NA. A dynamic network approach for the study of human phenotypes. PLoS Comput. Biol. [Internet]. 2009;5:e1000353. Available from: http://journals.plos.org/ploscompbiol/article?id=10.1371/journal.pcbi.1000353
