# Supplementary material for: From comorbidities of chronic obstructive pulmonary disease to identification of shared molecular mechanisms by data integration
Source: BMC Bioinformatics. 2016 Nov 22;17(Suppl 15):23–35. doi: 10.1186/s12859-016-1291-3 (PMC5133493; doi:10.1186/s12859-016-1291-3)
Supplement: Supplementary file 7 — Genes and Pathways relating COPD and Malignancies of Lower Respiratory Track (DG_5). The figure shows the association between genes (a) and (b, c) pathways for those ICD9 codes included in DG_5. A dark (light) blue square denotes that the association between disease and pathway or gene was computed as significant when using either mapping1_DG or mapping2_DG (only mapping1_DG). Selection criteria for the display of diseases, genes and gene-sets are the same as those described in Fig. 5. (PDF 1143 kb) [file 12859_2016_1291_MOESM7_ESM.pdf]

(a) Genes **(b) KEGG** **(c) Gene Ontology**

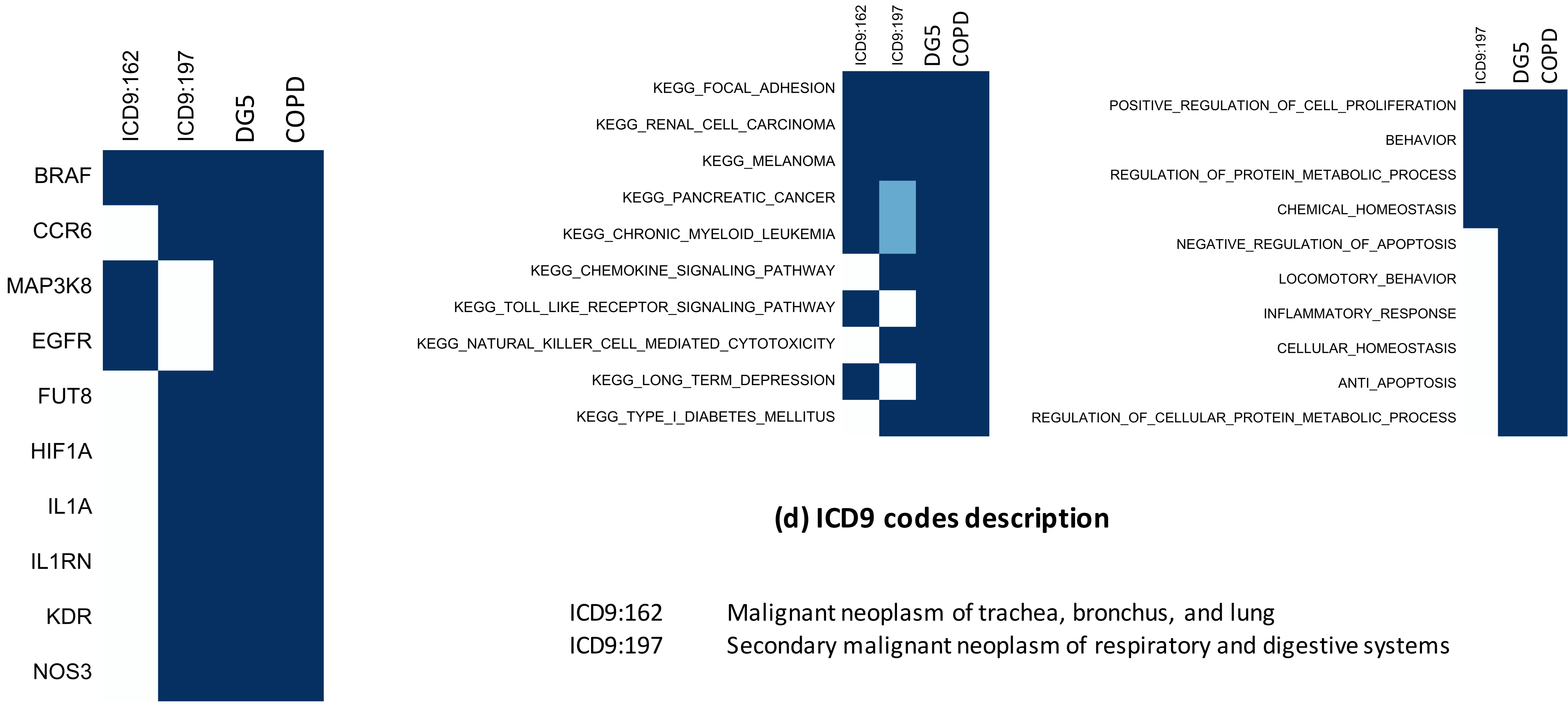

(d) ICD9 codes description

ICD9:162	Malignant neoplasm of trachea, bronchus, and lung
ICD9:197	Secondary malignant neoplasm of respiratory and digestive systems

Fig. S3
