# Supplementary material for: From comorbidities of chronic obstructive pulmonary disease to identification of shared molecular mechanisms by data integration
Source: BMC Bioinformatics. 2016 Nov 22;17(Suppl 15):23–35. doi: 10.1186/s12859-016-1291-3 (PMC5133493; doi:10.1186/s12859-016-1291-3)
Supplement: Supplementary file 9 — Genes and Pathways relating COPD and DG8. The figure shows the association between Reactome (a) and Biocarta (b) pathways for most-associated ICD9 codes included in DG8. A dark (light) blue square denotes that the association between disease and pathway or gene was computed as significant when using either mapping1_DG or mapping2_DG (only mapping1_DG). The description of the ICD9 codes is provided in panel (c). Additional file 9: Figure S8 extends the information provided in Fig. 5 and follows the same color-code and selection criteria. (PDF 1550 kb) [file 12859_2016_1291_MOESM9_ESM.pdf]

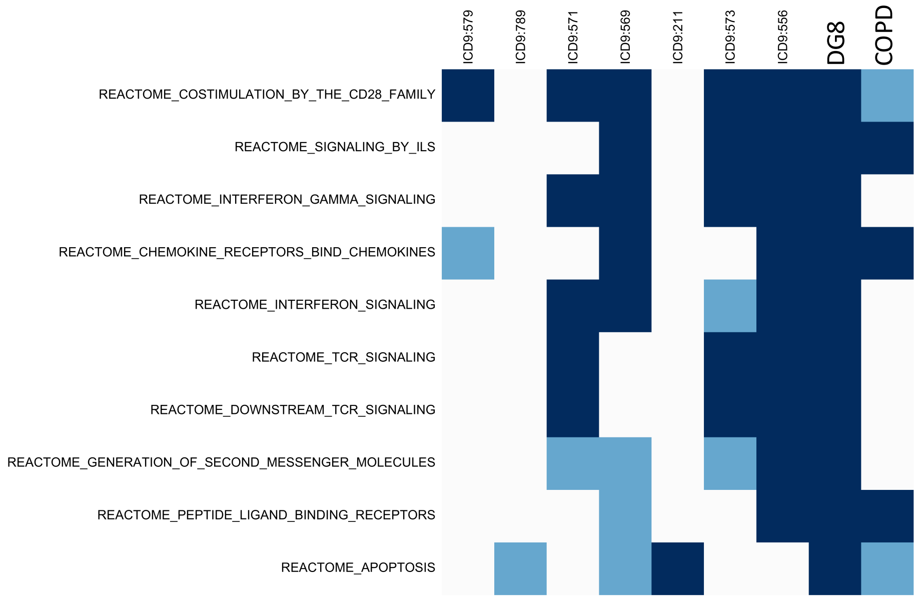

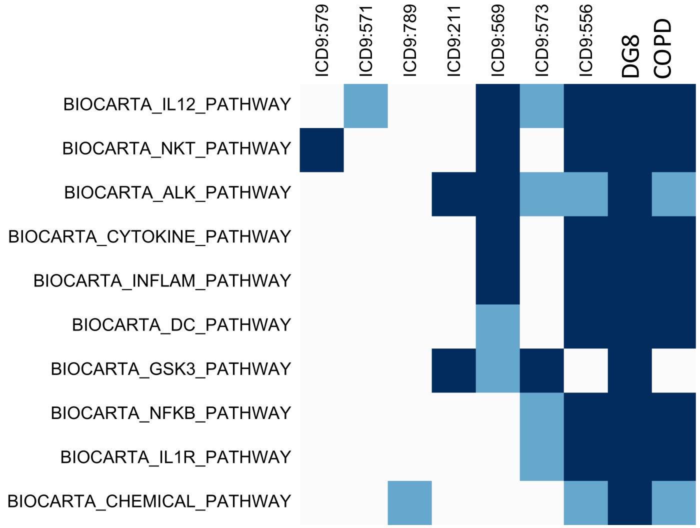

(c) ICD9 codes description

ICD9:211	Benign neoplasm of other parts of digestive system
ICD9:251	Other disorders of pancreatic internal secretion
ICD9:556	Ulcerative colitis
ICD9:569	Other disorders of intestine
ICD9:571	Chronic liver disease and cirrhosis
ICD9:573	Other disorders of liver
ICD9:577	Diseases of pancreas
ICD9:579	Intestinal malabsorption
ICD9:789	Other symptoms involving abdomen and pelvis

Fig. S5
